# A cross-sectional analysis of the PURE study on minerals intake among Malaysian adult population with hypertension

**DOI:** 10.1038/s41598-024-59206-0

**Published:** 2024-04-13

**Authors:** Nafiza Mat Nasir, Zaleha Md Isa, Noor Hassim Ismail, Rosnah Ismail, Azmi Mohd Tamil, Mohd Hasni Jaafar, Mohamed Syarif Mohamed Yassin, Najihah Zainol Abidin, Nurul Hafiza Ab Razak, Aziemah Zulkifli, Khairul Hazdi Yusof

**Affiliations:** 1 Department of Primary Care Medicine, Faculty of Medicine, Universiti Teknologi MARA (UiTM), Sungai Buloh Campus, Selangor Branch, Jalan Hospital, Sungai Buloh, Selangor Malaysia; 2 Department of Public Health Medicine, Faculty of Medicine, Universiti Kebangsaan Malaysia, Kuala Lumpur, Cheras Malaysia; 3 Department of Diagnostic and Allied Health Science, Faculty of Health and Life Sciences, Management and Science University, Shah Alam, Selangor Malaysia; 4 Institute for Environment and Development (LESTARI), Universiti Kebangsaan Malaysia, Bangi, Selangor Malaysia; 5 Risk Management Unit, Hospital Canselor Tuanku Muhriz, Universiti Kebangsaan Malaysia, Kuala Lumpur, Cheras Malaysia

**Keywords:** Micronutrients, Minerals, Dietary, Modification, High blood pressure, Health care, Risk factors

## Abstract

Hypertension (HPT) is the leading modifiable risk factor for cardiovascular diseases and premature death worldwide. Currently, attention is given to various dietary approaches with a special focus on the role of micronutrient intake in the regulation of blood pressure. This study aims to measure the dietary intake of selected minerals among Malaysian adults and its association with HPT. This cross-sectional study involved 10,031 participants from the Prospective Urban and Rural Epidemiological study conducted in Malaysia. Participants were grouped into HPT if they reported having been diagnosed with high blood pressure [average systolic blood pressure (SBP)/average diastolic blood pressure (DBP) ≥ 140/90 mm Hg]. A validated food frequency questionnaire (FFQ) was used to measure participants' habitual dietary intake. The dietary mineral intake of calcium, copper, iron, magnesium, manganese, phosphorus, potassium, sodium, and zinc was measured. The chi-square test was used to assess differences in socio-demographic factors between HPT and non-HPT groups, while the Mann–Whitney U test was used to assess differences in dietary mineral intake between the groups. The participants’ average dietary intake of calcium, copper, iron, magnesium, manganese, phosphorus, potassium, selenium, sodium, and zinc was 591.0 mg/day, 3.8 mg/day, 27.1 mg/day, 32.4 mg/day, 0.4 mg/day, 1431.1 mg/day, 2.3 g/day, 27.1 µg/day, 4526.7 mg/day and 1.5 mg/day, respectively. The intake was significantly lower among those with HPT than those without HPT except for calcium and manganese. Continuous education and intervention should be focused on decreasing sodium intake and increasing potassium, magnesium, manganese, zinc, and calcium intake for the general Malaysian population, particularly for the HPT patients.

## Introduction

Hypertension continues to be a public health problem despite many actions that have been taken to reduce its incidence across the world. According to the World Health Organization (WHO), an estimated 46% of adults with HPT are unaware that they have the condition, and of adults with HPT, only 1 in 5 (21%), approximately, have it treated (WHO, 2021). This multifactorial disease is responsible for more than 8 million deaths annually^1,2^.

Current evidence indicates that HPT is a multifactorial condition with genetic, sociodemographic, and behavioral risk factors, among others^2,3^. Some factors are non-modifiable, while others, such as physical activity and dietary intake, are well known modifiable risk factors. Individuals with HPT who adhered to their scheduled clinical sessions in a local public hospital were regularly advised to monitor their dietary intake in order to keep their blood pressure at an optimum level. They were also advised to reduce their salt intake and to include more vegetables and fruits in their diet.

Studies have shown a strong relationship between dietary intake and HPT, and several have proven the critical role of minerals (sodium, potassium, magnesium, zinc, calcium, selenium and copper) in food in maintaining an optimal blood pressure level^4–6^. Dietary sodium is a well-known risk factor of hypertension and there is strong association between salt and HPT^4^. There is significant evidence showing an association between calcium deficiencies and increases in blood pressure^7,8^. According to a recent longitudinal study, selenium might have an adverse impact on the development of HPT in the elderly^9^, and elevated selenium levels have been linked with increased levels of serum cholesterol^10,11^. A recent study has concluded that both deficiency and excessive dietary intake of iron would increase the risk of HPT^12^. Findings on associations between zinc, magnesium, manganese, phosphorus, potassium, selenium, copper and blood pressure are inconsistent^4,13–19^.

To the best of our knowledge, the current studies in Malaysia were focusing on the association between dietary sodium and potassium intake with hypertension^20,21^. Studies regarding association between other dietary minerals and hypertension are still scarce in Malaysia. This study aimed to determine the dietary intake of selected minerals (calcium, copper, iron, magnesium, manganese, phosphorus, potassium, selenium, sodium and zinc) among Malaysian adults and its association with HPT.

## Methodology

### Study design and population

This was a community-based study, a sub-study under the Prospective Urban Rural Epidemiology (PURE) study conducted among adults aged 35–70 years old. The PURE study involved 27 countries, including Malaysia that focused on the impact of societal influences on the prevalence of selected non-communicable diseases. The extensive methodology of the overall PURE study has been described in previous studies^22,23^.

### Measurements of dependent and independent variables

Blood pressure was measured by a trained research assistant using a calibrated Omron automatic digital monitor (Omron HEM-757; Omron Corp, Tokyo, Japan) after the participants had had 15 min of rest in a seated position. For purposes of this study, an individual with HPT is defined as one who reported having HPT and either (a) was receiving blood pressure-lowering medication or (b) had an average systolic blood pressure (SBP) of at least 140 mm Hg, or an average diastolic blood pressure (DBP) of at least 90 mm Hg, or both SBP and DBP that exceeded those levels. The readings of SBP and DBP were taken twice at five-minute intervals with appropriately sized cuffs based on a standard protocol. The average of the two readings was recorded and categorized as normal or HPT (SBP of 140 mm Hg or greater and/or DBP of 90 mm Hg or greater) according to the Malaysian Clinical Practice Guidelines (CPG) on Hypertension 2018^24^.

The dietary intake of participants was measured using a validated food frequency questionnaire (FFQ)^25^. A list of food and drink items was given to the participants, with pre-defined portion sizes and information on frequency of intake was sought with the following question: “During the past year, on average, how often have you consumed the following foods or drinks?” Responses ranged from “never” to “more than 6 times/day”. The dietary intake of each mineral—calcium, copper, iron, magnesium, manganese, phosphorus, potassium, selenium, sodium, and zinc—was estimated according to the recipes of dishes reported by the participants. To compute daily nutrient intake, this study utilized both the Malaysian Food Composition (MyFCD) database and the US Department of Agriculture (USDA) food-composition database, modifying them as necessary by incorporating information from local food-composition tables and nutrient databases that contained recipes for mixed dishes commonly consumed in the local area^26,27^.

Physical activity levels were captured using the International Physical Activity Questionnaire (IPAQ)^27–29^, and participants were also asked whether they had a medical diagnosis of other comorbidities such as diabetes mellitus (DM) and whether they had any family members with HPT and/or DM. Physical activity was classified as low if it was less than 600 Metabolic Equivalent (MET) minutes per week, and as high if it was equal to or greater than 600 MET minutes per week^30,31^.

Height and weight were measured using a portable stature meter and the TANITA (BC-558 Ironman®) segmental body composition analyzer, respectively. Body Mass Index (BMI) was calculated by dividing weight (in kilograms) by the square of height (in meters). Overweight was defined as a BMI equal to or greater than 25 kg/m^2^ but less than 30 kg/m^2^, and obesity was defined as a BMI equal to or greater than 30 kg/m^2^.

All the information was collected using a questionnaire developed by the Population Health Research Institute (PHRI). The questionnaire was later revised and validated by the Malaysian team of researchers to ensure its suitability for the local population. Face and content validity were carried out by the research team, which comprised experts in public health-related studies.

### Statistical analysis

The data were analyzed using SPSS version 26. The general characteristics are presented as frequencies and percentages (categorical data) and median with interquartile range (IQR) of continuous data. This study used the Recommended Nutrient Intakes (RNI) for selected minerals by the Ministry of Health (MOH) to examine whether participants meet their requirement. The chi-square test was used to assess the differences between HPT and non-HPT groups for the following variables: age, sex, location, education level, SES, employment status, marital status, BMI, physical activity, co-morbidity, a family history of HPT, and a family history of DM. The difference between the HPT groups for the dietary intake of minerals was assessed through the Mann–Whitney U test. The statistical significance level was set at *p* < 0.05.

### Ethical approval and consent to participate

All protocols were carried out in accordance with the relevant ethical guidelines and regulations. Each participant gave their informed written consents before taking part in the study. The Hamilton Health Sciences Research Ethics Board approved the study protocol (PHRI; Grant No. 101414), and local ethical clearance was obtained from the Research and Ethics Committee of Universiti Kebangsaan Malaysia (UKM) Medical Center (project code: PHUM-2012–01) and the Research Ethics Committee of Universiti Teknologi Mara (UiTM).

## Results

Table 1 shows the incidence of HPT, which was 43.5%. The majority of those with HPT were > 40 years old (48.0%), residing in rural areas (48.0%), having low education level (54.2%), and lower socio-economic status (48.2%). The majority of those with HPT were also currently unmarried (48.2%) and were overweight/obese (46.6%). Most of the respondents with HPT reported having co-morbidity of DM (68.4%) and a family history of HPT (48.7%) (Table 1).

**Table 1:** General characteristics of study participants (*n* = 10,031).

Characteristics	Hypertension, N (%)			*p* value
	Total	Yes	No	
	10,031	4365 (43.5)	5666 (56.5)	
Age (years)				
≤ 40	1514 (15.1)	274 (18.1)	1240 (81.9)	< **0**.001**
> 40	8517 (84.9)	4091 (48.0)	4426 (52.0)	
Sex				
Female	5682 (56.6)	2520 (44.4)	3162 (55.6)	0.054
Male	4349 (43.4)	1845 (42.4)	2504 (57.6)	
Location				
Urban	4797 (47.8)	1855 (38.7)	2942 (61.3)	< **0**.001**
Rural	5234 (52.2)	2510 (48.0)	2724 (52.0)	
Education level^#ϖ^				
Low	4242 (42.3)	2298 (54.2)	1944 (45.8)	< **0**.001**
High	5784 (57.7)	2064 (35.7)	3720 (64.3)	
SES^#^				
Low	3363 (35.7)	1622 (48.2)	1741 (51.8)	< **0**.001**
Middle-High	6067 (64.3)	2469 (40.7)	3598 (59.3)	
Employment status^#^				
Employed	5154 (51.7)	2234 (43.3)	2920 (56.7)	0.725
Unemployed	4813 (48.3)	2103 (43.7)	2710 (56.3)	
Marital status^#^				
Currently married	9039 (90.2)	3888 (43.0)	5151 (57)	**0**.002*
Single and/or widowed	980 (9.8)	472 (48.2)	508 (51.8)	
BMI				
Normal	3036 (30.3)	1106 (36.4)	1930 (63.6)	< **0**.001**
Overweight-Obese	6995 (69.7)	3259 (46.6)	3736 (53.4)	
Physical activity^#^				
Low	3477 (36.9)	1479 (42.5)	1998 (57.5)	0.205
High	5953 (63.1)	2612 (43.9)	3341 (56.1)	
Co-morbidity (DM)				
Yes	1695 (16.9)	1160 (68.4)	535 (31.6)	< **0**.001**
No	8336 (83.1)	3205 (38.4)	5131 (61.6)	
Family history (Hypertension)^#^				
Yes	3397 (34.04)	1653 (48.7)	1744 (51.3)	< **0**.001**
No	6582 (65.96)	2688 (40.8)	3894 (59.2)	
Family history (DM)^#^				
Yes	2474 (24.79)	1099 (44.4)	1375 (55.6)	0.304
No	7507 (75.21)	3246 (43.2)	4261 (56.8)	

*Significant at *p* value  < 0.05; **significant at *p* value  < 0.001; ^#^the total numbers (*n*) are not equal to 10,031 due to missing values; ^ϖ^Low educational level refers to “none and primary level” while high educational level represented by “secondary and tertiary level”. Significant values are in bold.

Comparison of participants dietary intake of minerals with the Recommended Nutrient Intakes (RNI) for selected minerals by the Ministry of Health (MOH) are reported in Table 2. The average intakes of calcium, copper, iron, magnesium, manganese, phosphorus, potassium, selenium, sodium, and zinc were 591 mg/day, 3.8 mg/day, 27.1 mg/day, 32.4 mg/day, 0.41 mg/day, 1431 mg/day, 2.4 g/day, 27.1 µg/day, 4526 mg/day and 15 mg/day, respectively. For copper, phosphorus, and sodium, the dietary intake of these minerals was reported to be higher than RNI, especially sodium, which was three times higher. The dietary intake among participants were lower than the RNI for calcium, magnesium, manganese, potassium, and zinc. However, for iron and selenium, there were slight differences in the minerals' intake according to sex and age groups. Among those with HPT, the dietary intake of all the minerals except for calcium and manganese was significantly lower as compared to those without HPT.

**Table 2:** Intake of dietary trace elements among Malaysian adults (*n* = 10,031).

Trace elements	Recommended Nutrient Intakes (RNI) 2017^α^			Intake of dietary trace elements among study population			
	Age groups	Sex		Hypertension			Mann–Whitney U * p* value
		Male	Female	Med (IQR: 25th–75th percentile)			
				Overall	Yes	No	
Calcium (mg/day)	30–50	1000	1000	591.0 (387.8–814.7)	583.1 (380.9–809.2)	594.8 (393.3–817.5)	0.067
	51–59		1200				
	60–65						
	> 65						
Copper (mg /day)	30–50	0.9	0.9	3.8 (0.29–8.17)	3.78 (0.14–8.14)	3.87 (1.02–8.18)	< **0**.001**
	51–59						
	60–69						
	≥ 70						
Iron (mg/day)	30–50	14	29	27.1 (18.3–39.5)	26.2 (17.4–38.7)	27.8 (19.1–40.2)	< **0**.001**
	51–59		11				
	60–65						
	> 65						
Magnesium (mg/day)	30–50	420	320	32.4 (17.8–59.6)	29.9 (15.4–56.9)	34.9 (19.5–65.4)	< **0**.001**
	51–59		420				
	60–69						
	≥ 70		320				
Manganese (mg/day)	30–50	2.3	1.8	0.4 (0.2–0.7)	0.4 (0.2–0.7)	0.4 (0.2–0.7)	**0**.**514**
	51–59						
	60–69						
	≥ 70						
Phosphorus (mg/day)	30–50	700	700	1431.1 (953.0–1992.0)	1411.5 (925.0–1975.6)	1451.7 (974.8–2015.7)	**0**.002*
	51–59						
	60–69						
	≥ 70						
Potassium (g/day)	30–50	4.7	4.7	2.3 (1.6–3.4)	2.3 (1.5–3.3)	2.4 (1.6–3.4)	< **0**.001**
	51–59						
	60–69						
	≥ 70						
Selenium (µg/day)	30–50	32	24	27.1 (14.9–47.0)	25.3 (13.5–44.9)	28.8 (16.3–49.2)	< **0**.001**
	51–59						
	60–65	31	23				
	> 65	30					
Sodium (mg/day)	30–50	1500	1500	4526.7 (2835.6–7646.4)	4229.5 (2656.9–6843.2)	4757.5 (2927.1–8526.3)	< **0**.001**
	51–59						
	60–69						
	≥ 70	1200	1200				
Zinc (mg/day)	30–50	6.5	4.6	1.5 (0.9–2.5)	1.4 (0.8–2.4)	1.6 (1.0–2.5)	< **0**.001**
	51–59						
	60–65	6.3	4.4				
	> 65	6.2	4.3				

*Significant at *p* value < 0.05; **significant at *p* value < 0.001; ^**α**^RNI by Ministry of Health (MOH). Significant values are in bold.

## Discussion

This study has shown that 43.5% of the participants had HPT. The findings revealed that this population had higher intake of sodium compared to RNI and lower intake of potassium and calcium, which are important for regulating blood pressure. In contrast, the intake of copper, iron, phosphorus, and selenium was higher than RNI among all participants, regardless of HPT status. Excessive intake of copper, iron, selenium, and phosphorus was associated with increased risk of HPT. The study also highlighted the higher incidence of HPT among older individuals, those who were overweight or obese, and those with a history of diabetes or family history of HPT or diabetes.

The incidence of HPT among this study population was 43.5%, which was higher than the incidence reported in the three consecutive NHMS reports for 2011 (32.7%), 2015 (30.3%), and 2019 (30.0%)^24,32,33^. This difference was expected, however, since NHMS covered individuals aged 18 years and above whereas this study limited its participants to adults 35 to 70 years old. The age range of 35 years and above is known to have a higher risk of NCDs, including HPT. This study found that individuals older than 40 years were more likely to have HPT than the younger participants (48.0% vs 18.1%). This may be caused by arterial stiffening due to aging, which is also closely associated with the progression of cardiovascular disease (CVD)^34,35^. Limited access to healthcare and low health literacy may have caused the late detection of these individuals’ health conditions, especially among less educated, those with low socioeconomic status, or residing in rural areas^36^. Additionally, HPT was more common among overweight or obese participants, those with history of diabetes or having family history of HPT or diabetes. Population-based studies have suggested that two-thirds of HPT can be directly attributed to obesity^37,38^. In addition to genetic factors, lifestyle choices (diet or food choices and physical activity levels), which tend to be similar among family members, have been demonstrated to be a strong risk factor for HPT^39^.

The findings highlighted the sodium intake of this population (4526 mg/day) was three times higher than the RNI (1500 mg/day), regardless of the HPT status. The sodium intake by this population is similar to the China population (4505 mg/day) but higher than the US population (3232 mg/day)^40,41^. However, this study showed that the sodium intake among those with HPT (4229.5 mg/day) was significantly lower compared to those without HPT (4757.5 mg/day). The finding is concurrent with a study done in the US that reported HPT and non-HPT groups consumed 3246.1 mg/day and 3410.1 mg/day of sodium respectively^42^. Sodium in the form of table salt, which is a common flavor enhancer widely used in cooking and processed and packaged foods, sauces, and snacks, is thought to be the cause of 40–50% of all types of HPT^20,43^. High consumption of sodium can trigger endogenous cardiotonic steroids (CTs), which act as Na+/K+ pump inhibitors^44^. These CTs may cause sodium retention (Na+ overload) and alter the vascular tone, causing damage to the endothelium, leading to arterial stiffness, and increasing the risk of HPT.

This study found those with HPT had a significantly lower intake of potassium (2.3 g/day) compared to those without HPT (2.4 g/day). The US population also shows similar patterns, National Health and Nutrition Examination Survey (NHANES) from 2007 to 2014 reported that potassium intake among HPT and non-HPT were 2.3 g/day and 2.5 g/day respectively^42^. Several studies have suggested a low dietary intake of potassium, may also lead to HPT. Potassium, which plays a vital role in cellular metabolism as well as electrolyte and fluid balance, affects blood pressure in a manner contrary to sodium due to the action of the sodium–potassium pump^45^. Adequate potassium intake aids in exerting hypotensive activity by suppressing the sympathetic nervous system (SNS). This leads to lower production of renin and angiotensin-II by inhibiting angiotensin-converting enzyme (ACE) and by acting as an angiotensin receptor blocker (ARB)^46^; as a result, it promotes better regulation of blood volume and cardiac output (CO). A sufficient intake of potassium also helps stimulate adenosine triphosphate (sodium/potassium ATPase), which promotes sodium excretion and results in decreased blood pressure^47^. Therefore, an adequate dietary intake of potassium is crucial in a daily diet, especially among those with high-sodium diets.

Although this study does not show any significant difference of dietary calcium intake between the HPT groups, the low dietary intake of calcium may hinder the ability of intracellular calcium ions to effectively regulate vascular tone, thereby affecting the blood pressure^7,48^. The calcium ion helps to enhance diuretic action, which aids in sodium secretion, encouraging better regulation of blood volume and CO via activation of SNS^49^. The low calcium intake found in this population was in line with previous studies, which reported dietary calcium intake of between 357 and 397.2 mg/day^50–52^. It was also supported by the findings of an International Osteoporosis Foundation (IOF) research committee, which concluded that countries from South, East, and Southeast Asia have the world’s lowest average calcium intake, which is often less than 400 mg/day^53^.

For copper, iron, phosphorus, and selenium, the dietary intake reported by all study participants was higher than the RNI. Similarly, the US population also consumed dietary copper, iron, phosphorus, and selenium higher than the recommended dietary allowances (RDA) by the WHO^54^. Although the intakes were much lower among those with HPT, they were still higher than the recommended values. Too much copper is said to suppress myosin-ATPase activity, which will lead to calcium overload, later resulting in elevated blood pressure^17^. Excessive iron intake has been shown to play a role in the development of HPT, increasing free radicals and oxidative stress, which leads to endothelial damage and the development of HPT^55–57^. As for selenium, too much of this mineral can adversely affect the major organs due to its pro-oxidant activity, which can disrupt the normal regulation of blood pressure^58^. Studies have also shown that excessive phosphorus intake activates the sympathetic nervous system, which accelerates cardiac activity and increases blood pressure^59^.

The intake of magnesium, manganese, and zinc among this study population was much lower than the recommended values. In contrast, the US population was reported to have dietary zinc intake higher than the RDA and the German population was reported to have adequate dietary manganese as recommended by the societies for nutrition in the region^54,60^. Studies have long linked the deficiency of these minerals with HPT^4,61–63^. Magnesium, which has an antiarrhythmic effect, may influence blood pressure levels by modulating the vascular tone^62^. Low extracellular manganese levels can adversely affect the production and release of nitric oxide (NO), resulting in the alteration of arterial smooth muscle tone leading to HPT^14,64^. As for zinc, its deficiency may elevate blood pressure by altering vascular tone^63^. Moreover, inadequate zinc intake is said to alter an individual’s taste sensitivity to salt, causing increased salt consumption, which is known to promote high blood pressure^61^.

The overall dietary intake of all the minerals except for calcium and manganese was significantly lower among those with HPT. Since this was a cross-sectional study, the temporal link between dietary intake and the occurrence of HPT could not be determined. Thus, the lower dietary intake among the respondents with HPT could potentially be explained by dietary modifications that the individuals made after having been diagnosed with HPT^65–67^. Without a proper understanding of the guidelines on diet modification, some individuals tend to reduce all types of food groups following an HPT diagnosis^68^. This can lead to an inadequate intake of some minerals that play a vital role in maintaining optimum blood pressure. Regardless of the insignificant difference of calcium intake between HPT and non-HPT groups, the overall intake of all participants was lower than the RNI level. It may be due to Asian cultural dietary habits, which commonly involve non-dairy diets^69,70^.

This study involved more than 10,000 participants from rural and urban areas of Malaysia who thoroughly reported their dietary intakes. Thus, the dietary intake of this study’s participants provided grounds for discussing the deficiency and overconsumption of minerals. The study’s main limitation was that dietary intakes was self-reported, which may have potentially overestimated or underestimated the dietary intakes of the respondents due to recall bias. Furthermore, the quantification of the minerals was calculated based on the meals that the respondents reported having consumed. This may be subject to slight inaccuracy since the calculation was based on general recipes for each dish. However, this study only included participants with plausible energy intake in the range of 500–5000 kcal to control the over- or underestimation of dietary intakes. Also, it is encouraged that future studies to analyze biomarkers of minerals in blood serum for a more accurate interpretation of the nutritional status of the study population.

## Conclusion

Increased dietary intake of certain minerals, especially potassium, magnesium, manganese, zinc, and calcium (low in this population), as well as reduced intake of sodium and selenium, could positively modulate BP levels, thereby lowering the risk of HPT. Continuous professional education among doctors should be promoted to increase awareness and knowledge of the role these minerals play in common conditions such as hypertension. Public health campaigns to increase awareness of the importance of consuming an adequate amount of these minerals should also be carried out.

## Data Availability

The data that support the findings of this study are available from PHRI but restrictions apply to the availability of the data, which were used under license for the current study, and are not publicly available. Data are however available from the authors upon reasonable request and with permission from PHRI.

## Abbreviations

PURE: Prospective Urban and Rural Epidemiological Study
HPT: Hypertension
CVDs: Cardiovascular diseases
SBP: Systolic blood pressure
DBP: Diastolic blood pressure
FFQ: Food frequency questionnaire
WHO: World Health Organization
MyFCD: Malaysian Food Composition Database
USDA: United States Department of Agriculture
IPAQ: International Physical Activity Questionnaire
MET: Metabolic equivalent
BMI: Body mass index
PHRI: Population Health Research Institute
IQR: Interquartile range
RNI: Recommended nutrient intakes
RDA: Recommended dietary allowances
MOH: Ministry of Health
DM: Diabetes mellitus
